# Efficacy and safety of robotic liver surgery for the elderly: A propensity‐score matched analysis of short‐term outcomes with open liver surgery at a single center in Denmark

**DOI:** 10.1002/jhbp.12015

**Published:** 2024-06-12

**Authors:** Daisuke Fukumori, Christoph Tschuor, Takashi Hamada, Luit Penninga, Jens Hillingsø, Lars Bo Svendsen, Peter Nørgaard Larsen

**Affiliations:** ^1^ Department of Surgery and Transplantation, Rigshospitalet Copenhagen University Hospital Copenhagen Denmark; ^2^ CAMES, University of Copenhagen Copenhagen Denmark

**Keywords:** elderly patients, postoperative complications, propensity score matching, robotic liver surgery, short‐term outcomes

## Abstract

**Background:**

The incidence of liver tumors requiring surgical treatment continues to increase in elderly patients. This study compared the short‐term results of robotic liver surgery (RLS) versus open liver surgery (OLS) for liver tumors in elderly patients.

**Methods:**

A prospective database including all patients undergoing liver surgery at Copenhagen University Hospital between July 2019 and July 2022 was managed retrospectively. Short‐term surgical outcomes of the two main cohorts (OLS and RLS) and subgroups were compared using propensity score matching (PSM) in elderly patients (age ≥ 70 years) with liver tumors.

**Results:**

A total of 42 matched patients from each group were investigated: the RLS group had significantly larger tumor diameters, less blood loss (821.2 vs. 155.2 mL, *p* < .001), and shorter hospital stays (6.6 vs. 3.4 days, *p* < .001). Overall morbidity was comparable, while operative times were longer in the RLS group. The advantages observed with the robotic approach were replicated in the subgroup of minor liver resections.

**Conclusions:**

In patients ≥70 years, RLS for liver tumors results in significantly less blood loss and shorter hospital stays than OLS. RLS, especially minor liver resection, is safe and feasible in elderly patients with liver tumors.

## Graphical abstract

Fukumori and colleagues compared the short‐term results of robotic liver surgery with those of conventional open liver surgery in elderly patients aged 70 years and over and used propensity score‐matched analysis to assess safety and efficacy. Robotic liver surgery can be safely performed in patients aged 70 years and older.

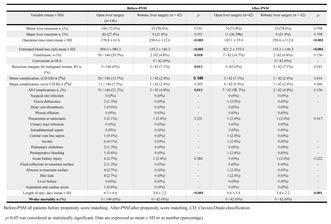

## 1 INTRODUCTION

In recent years, increased life expectancy and advances in treatments for chronic diseases have led to increased opportunities for treating the elderly population. The elderly suffer from age‐related frailty, metabolic decline, and complications of the heart, lungs, kidneys, and other organs. Open liver surgery (OLS) is considered a safe procedure for elderly patients, with acceptable levels of postoperative morbidity and mortality. However, it is important to note that the risk of complications, such as postoperative pneumonia, remains high and poses a potentially life‐threatening concern in this population^1^, ^2^, ^3^

Laparoscopic liver resection (LLS) was first reported in 1991,^4^ and since then, its surgical outcomes and techniques have been compared and reported to be less invasive than OLS, with similar surgical and oncological outcomes.^5^, ^6^ Furthermore, several reports of LLS in the elderly have shown it to be safe and effective, and when performed in patients ≥70 years, the results are comparable to, or better than, OLS.^7^, ^8^, ^9^ These studies have prompted LLS to become the procedure of choice in recent years for elderly patients.

Robot‐assisted liver surgery (RLS) was first introduced in 2003 by Giulianotti et al.^10^ A systematic review and meta‐analysis of RLS and conventional LLS has since concluded that the techniques are equivalent.^11^ Many studies report that a consensus is developing on the safety of RLS, with more surgeons electing for the robotic approach. However, studies specifically examining the application of RLS in the elderly are still limited, and the true benefits in this population remain unclear. The objective of the present study was therefore to compare the short‐term outcomes of RLS and OLR in elderly patients.

## 2 METHODS

Currently, approximately 400 liver surgeries are performed annually at our institution, and since 2009, 10% to 15% of these surgeries have been performed by LLS. The robotic system has several advantages over conventional laparoscopic surgery, including 7° of freedom when using the EndoWrist technology, the ability to reach posterior superior lesions, improved suturing ability, elimination of physiological tremors, and superior surgeon ergonomics. Despite the downside of additional costs and longer operative time compared to the laparoscopic approach, the aforementioned advantages and strong confidence in this new technology led to the decision to introduce RLS starting in 2019. Therefore, LLS was not performed after the introduction of RLS.

With regard to liver function, patients considered for liver resection in our center must have a Child‐Pugh score class A. The indications for OLS are (i) the absence of clinically significant portal hypertension and (ii) sufficient residual liver volume with respect to the condition of the liver parenchyma. The indications for RLS are initially tumor diameter less than 5 cm, sufficient residual liver volume, no portal hypertension, no anatomical variations, and good health status. With increasing experience, the indications have been extended to patients with larger tumors, multiple tumors, and anatomical variations.

**FIGURE 1:**
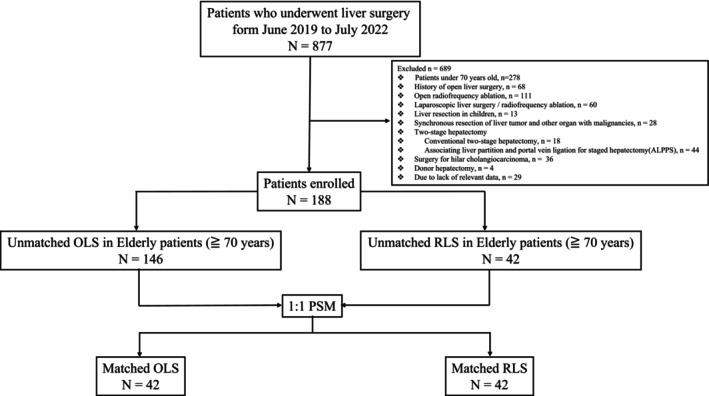
Short‐term results of robotic liver surgery (RLS) with conventional open liver surgery in elderly patients aged 70 years and over and propensity‐matched score analysis were used to assess safety and efficacy. Our results showed that RLS can be safely performed in patients aged 70 years and older.

All patients aged ≥70 years were included in the study. A retrospective study was conducted on 188 consecutive patients aged ≥70 years who underwent liver surgery at our center from November 2019 to July 2022 (Figure 1). Using the da Vinci Surgical System Si (Intuitive Surgical, Sunnyvale, California, USA), the short‐term outcomes of 42 patients undergoing RLS were compared to the short‐term outcomes of 146 patients undergoing OLS. The cohort was further divided into two subgroups by minor and major resection for analysis. For comparative analysis, all patients were matched 1:1 by PSM for the two main cohorts (OLS and RLS) and subgroups. PMS considered age, gender, body mass index (BMI), American Society of Anesthesiologists (ASA), and diagnosis as primary confounders in the assignment of OLS and RLS.

Indications for surgery included malignant and benign liver tumors. Malignant tumors included hepatocellular carcinoma, intrahepatic cholangiocarcinoma (iCC), gallbladder cancer, colorectal liver metastases (CRLM), and noncolorectal liver metastases. Benign tumors included hemangiomas, adenomas, and focal nodular hyperplasia. For microscopic distance of resection margins, if no tumor cells were present on the resection surface or within 1 mm, the resection margin was considered ontologically R0; however, if tumor cells were present on or within 1 mm of the resection surface, the resection margin was microscopically R1. Clinical factors examined included demographics, IWATE criteria, surgical outcomes, and postoperative results. Estimated blood loss (EBL) was obtained from anesthesia records. Operative time (OT) was defined as the time from skin incision to wound closure. No adjustment was made for debridement; postoperative complications occurring within 30 days were classified according to the Clavien‐Dindo (CD) classification. Major complications were defined as events requiring surgical, endoscopic, or radiological intervention (CD grade ≥ III).

### 2.1 Resection type and definitions

Minor liver resection was defined as a Couinaud's resection of ≤2 segments. Major liver resections were defined as the resection of ≥3 Couinaud segments.^12^ In addition, subsegmentectomy and monosegmentectomy were defined according to Wakabayashi et al.^13^

### 2.2 Exclusion criteria

Exclusion criteria included: (1) general pneumoperitoneum contraindications, cardiac or respiratory failure, and/or ASA physical status ≥III; (2) patients <70 years; (3) history of OLS; (4) open radiofrequency ablation; (5) LLS/laparoscopic radiofrequency ablation; (6) liver resection in children; (7) synchronous resection of liver tumor and another organ with malignancies; (8) two‐stage hepatectomy (conventional two‐stage hepatectomy and associated liver partition and portal vein ligation for staged hepatectomy [ALPPS]); (9) surgery for hilar cholangiocarcinoma; (10) donor hepatectomy and (11) due to lack of relevant data (Figure 1).

### 2.3 Operative technique for RLS

Robotic liver surgery was performed using only the Robotic Harmonic scalpel (Ethicon Endosurgery, Inc.), for liver parenchymal dissection (no use of the Cavitron ultrasound surgical aspirator [CUSA]).^14^ Pneumoperitoneal pressure was established at 8–12 mmHg and was maintained using an AirSeal system (SurgiQuest Inc., Milford, Connecticut, USA). The EndoWrist function of the Maryland bipolar forceps and fenestrated bipolar forceps are effective for controlling minor bleeding and dissecting and isolating major vessels. Intraoperative ultrasound was routinely used to ascertain tumor location and exclude additional tumors in the liver remnant. For RLS, an initial transparenchymal Glissonian approach is performed,^14^ in which a preliminary one‐way liver parenchymal dissection from the caudal or dorsal side to the deep part of liver is made, exposing the root of the target Glissonean pedicle intrahepatically. Identification of the target Glissonian pedicle was performed by the console surgeon, using an intraoperative ultrasound probe, to give sight of both the surgical field and a fully robotic ultrasound examination in real time, thus clarifying the surrounding anatomy and allowing detection and sequential dissection of tumors. Blood vessels or bile ducts with a diameter of >3 mm were ligated using Weck Hem‐o‐lok clips with a dorsal approach. Both the Glissonean pedicle and the major hepatic vein >10 mm were transected intrahepatically using a laparoscopic Endo‐GIA stapler with a dorsal approach introduced by the bedside surgeon. Pringle's maneuver was used for anatomical liver resection of more than one segment. During transection of the liver parenchyma, central venous pressure <5 mmHg was maintained to prevent venous hemorrhage. Pneumoperitoneal pressure during hemostasis assessment was set at 8 mmHg. After confirming no bile leakages or bleeding from the resection margin, surgery was ended.

### 2.4 Operative technique for OLS

The abdominal incision consisted of a right‐angle laparotomy in the right upper quadrant, allowing favorable access to the posterior liver segments. The liver was mobilized by a dissection of the round, falciform ligaments and ipsilateral coronary and triangular ligaments. Intraoperative ultrasonography was used to plan the parenchymal dissection, and resection lines were delineated on the liver surface with electrocautery marks. Dissection of the liver parenchyma was performed using an ultrasonic harmonic scalpel (Ethicon EndoSurgery) or LigaSure Dolphin tip open sealer/divider and a water‐jet dissector (courtesy of ERBE USA) or CUSA. Vessels <3 mm in diameter were cut with an ultrasonic harmonic scalpel (Ethicon EndoSurgery) or LigaSure Dolphin Tip open dealer/divider. Vessels >3 mm in diameter were ligated or closed with a LIGACLIP. The Pringle maneuver was selectively used depending on the case. Inflow occlusion was applied intermittently, with 15 min of occlusion alternating with 5 min of reperfusion. During transection of the liver parenchyma, the central venous pressure was maintained <5 mmHg to prevent venous hemorrhage. The few larger vessels and portal triads were divided using an endoscopic stapler (Endo‐Gia Stapler; Covidien, Norwalk, Connecticut, USA). An argon beam coagulator was used to refine unsatisfactory hemostasis, possibly in addition to the application of hemostatic matrixes on the transection surface. A closed suction drain was used as an abdominal drainage tube.

### 2.5 Postoperative care

All liver resection patients underwent the enhanced recovery after surgery program (ERAS) as previously published.^15^

### 2.6 Statistical analysis

Statistical analyses were performed using IBM SPSS version 26, with Fisher's exact test for categorical data and student's *t*‐test for continuous data. In addition, a PSM analysis was performed to control for selection bias. A 1:1 PSM analysis was performed using the nearest‐neighbor matching method to minimize baseline characteristic differences between RLS and OLS. Patient baseline characteristics (age, gender, BMI, ASA grade), pathology, and operative procedure were included in propensity scores. Nearest neighbor matching without replacement was performed, with the caliper set at 0.119. Continuous variables with normal distribution are expressed as mean ± standard deviation or median and categorical variables are expressed as number (*n*) or proportion (%). Variables with normal distribution were tested by the student's *t*‐test, whereas variables without normal distribution were tested by the Mann–Whitney U‐test. Categorical variables were analyzed using the chi‐squared test or Fisher's exact test. A *p*‐value of < .05 was considered statistically significant.

## 3 RESULTS

A total of 188 patients aged ≥70 years received RLS or OLS between November 2019 and July 2022. Of these, 42 patients were allocated to the RLS group and 146 to the OLS group. After PSM, the RLS and OLS groups were matched 1:1, with 42 patients assigned to each. The demographics and outcomes of RLS and OLS, before and after PSM, are summarized in Table 1. Before PSM gender, BMI, ASA score, and histological diagnosis did not differ significantly. There was a trend toward a significantly higher history of previous laparotomy in the OLS group. No significant differences were found between the two groups with regard to tumor size. After PSM, no significant differences in median age or gender distribution were identified between RLS and OLS. There were also no significant differences in mean BMI, ASA, or history of abdominal surgery. According to the IWATE criteria, the score was 5.6 ± 2.5 in RLS, of which 19 (45.2%) were judged as high advanced/expert. In terms of tumor size, there was a significant difference between the two groups (42.8 mm for RLS vs. 30.9 mm for OLS, *p* = .004), with significantly larger tumors in RLS.

**TABLE 1:** Demographics and baseline characteristics of elderly patients.

Variable	Before‐PSM		*p*‐value	After‐PSM		*p*‐value
	Open liver surgery (*n* = 146)	Robotic liver surgery (*n* = 42)		Open liver surgery (*n* = 42)	Robotic liver surgery (*n* = 42)	
Age, years, mean ± SD	75.3 ± 4.1	77.5 ± 4.9	**.003**	76.5 ± 4.6	77.5 ± 4.9	.337
Sex, male, *n* (%)	97 (66.4%)	27 (64.3%)	.854	26 (61.9%)	27 (64.3%)	1.000
BMI (kg/m2), mean ± SD	26.5 ± 4.9	25.8 ± 3.9	.447	27.2 ± 5.3	25.8 ± 3.9	.202
ASA‐score			.556			.370
I	0 (0%)	0 (0%)	‐	0 (0%)	0 (0%)	‐
II	60 (41.1%)	14 (33.3%)	.474	18 (42.9%)	14 (33.3%)	.501
III	85 (58.2%)	28 (66.7%)	.374	23 (54.8%)	28 (66.7%)	.372
IV	1 (0.7%)	0 (0%)	1.000	1 (2.4%)	0 (0%)	1.000
Diagnosis			.328			.639
CRLM	79 (54.1%)	17 (40.5%)	.161	24 (57.1%)	17 (40.5%)	.190
HCC	32 (21.9%)	11 (26.2%)	.540	9 (21.4%)	11 (26.2%)	.798
iCC	17 (11.6%)	6 (14.3%)	.603	3 (7.1%)	6 (14.3%)	.483
Gallbladder cancer	6 (4.1%)	4 (9.5%)	.234	4 (9.5%)	4 (9.5%)	1.000
Benign	4 (2.7%)	3 (7.1%)	.187	1 (2.4%)	3 (7.1%)	.616
Other	8 (5.5%)	1 (2.4%)	.686	1 (2.4%)	1 (2.4%)	1.000
IWATE criteria: mean ± SD	‐	5.6 ± 2.5	‐	‐	5.6 ± 2.5	‐
Previous abdominal surgery						
Laparotomy *n* (%)	5 (3.4%)	5 (11.9%)	**.046**	1 (2.4%)	5 (11.9%)	.202
Laparoscopy *n* (%)	67 (45.9%)	18 (42.9%)	.861	24 (57.1%)	18 (42.9%)	.275
Tumor size, mm, mean ± SD	36.5 ± 31.0	42.8 ± 32.8	.260	30.9 ± 16.6	42.8 ± 32.8	**.040**

*Note*: Before‐PSM, all patients before propensity score matching. After‐PSM, after propensity score matching. Abbreviations: ASA, American Society of Anesthesiologists; BMI, body mass index; CRLM, colorectal liver metastasis; HCC, hepatocellular carcinoma; iCC intrahepatic cholangiocarcinoma; PSM, propensity score matching; SD, standard deviation. Data are expressed as mean ± SD or as numbers (percentage). *p* < .05 was considered statistically significant. Bolded values indicates statistically significant differences.

The resection types are summarized in Table 2. In terms of surgical methods, there were no significant differences between the RLS and OLS groups for each resection type before and after the PSM.

**TABLE 2:** Resection type.

Variable	Before‐PSM		*p*‐value	After‐PSM		*p*‐value
	Open liver surgery (*n* = 146)	Robotic liver surgery (*n* = 42)		Open liver surgery (*n* = 42)	Robotic liver surgery (*n* = 42)	
Subsegmentectomy *n* (%)	60 (41.1%)	12 (28.6%)	.154	19 (45.2%)	12 (28.6%)	.174
Monosegmentectomy *n* (%)	26 (17.8%)	8 (19.0%)	.823	6 (14.3%)	8 (19.0%)	.771
Bisegmentectomy *n* (%)	18 (12.3%)	13 (31.0%)	.008	6 (14.3%)	13 (31.0%)	.116
Right posterior sectionectomy/extended right posterior sectionectomy *n* (%)	2 (1.4%)	0 (0%)	1.000	0 (0.0%)	0 (0.0%)	‐
Central liver resection *n* (%)	0 (0%)	1 (2.4%)	.223	0 (0.0%)	1 (2.4%)	1.000
Left hemi‐hepatectomy/extended left hemi‐hepatectomy *n* (%)	25 (17.1%)	6 (14.3%)	.815	9 (21.4%)	6 (14.3%)	.570
Right hemi‐hepatectomy/extended right hemi‐hepatectomy *n* (%)	15 (10.3%)	2 (4.8%)	.369	2 (4.8%)	2 (4.8%)	1.000

*Note*: Before‐PSM, all patients before propensity score matching. After‐PSM, after propensity score matching. Abbreviation: PSM, propensity score matching; SD, standard deviation. Data are expressed as mean ± SD or as numbers (percentage). *p* < .05 was considered statistically significant.

Perioperative results are summarized in Table 3. After PSM, the numbers of minor and major liver resections were not significantly different between the RLS and OLS groups. OT tended to be significantly longer in RLS (256.6 vs. 165.1 min, *p* < .001). In terms of EBL, this was significantly less in the RLS group compared with the OLS group (148.9 vs. 683.5 mL, *p* < .001), while intraoperative blood transfusion rates were comparable to OLS (4.8% vs. 16.7%, *p* = .156). Regarding resection margin (R1) rate of malignant lesions, there was an insignificant trend toward higher R1 in RLS (7.1% vs. 0%, *p* = .241). There were no significant differences in postoperative complication rates between the two groups for minor complications (CD grade I–II), major complications (CD grade III–V), or overall complication rates. In terms of length of stay (LOS), mean LOS for RLS tended to be significantly shorter than for OLS (3.4 vs. 6.6 days, *p* < .001). Ninety‐day mortality was not observed between the two groups.

**TABLE 3:** Perioperative outcomes of elderly patients.

Variable (mean ± SD)	Before‐PSM		*p*‐value	After‐PSM		*p*‐value
	Open liver surgery (*n* = 146)	Robotic liver surgery (*n* = 42)		Open liver surgery (*n* = 42)	Robotic liver surgery (*n* = 42)	
Minor liver resection *n* (%)	106 (72.6%)	33 (78.6%)	.551	31 (73.8%)	33 (78.6%)	.798
Major liver resection *n* (%)	40 (27.4%)	9 (21.4%)	.551	11 (26.2%)	9 (21.4%)	.798
Operation time (min) mean ± SD	178.8 ± 61.6	256.6 ± 112.6	**<.001**	165.1 ± 55.0	256.6 ± 112.6	**<.001**
Estimated blood loss (mL) mean ± SD	994.0 ± 980.3	155.2 ± 146.3	**<.001**	821.2 ± 719.4	155.2 ± 146.3	**<.001**
Transfusion, *n* (%)	30/146 (20.5%)	2/42 (4.8%)	**.018**	7/42 (16.7%)	2/42 (4.8%)	.156
Conversion to OLS	‐	0/42 (0%)	‐	‐	0/42 (0%)	‐
Resection margins for malignant lesions, R1 *n* (%)	0/146 (0%)	3/42 (7.1%)	**.011**	0/42 (0%)	3/42 (7.1%)	.241
Minor complication (CD I/II) *n* (%)	20/146 (13.7%)	1/42 (2.4%)	**.049**	3/42 (7.1%)	1/42 (2.4%)	.616
Major complication (over CD III) *n* (%)	11/146 (7.5%)	1/42 (2.4%)	.305	4/42 (9.5%)	1/42 (2.4%)	.360
All complications *n* (%)	31/146 (21.2%)	2/42 (4.8%)	**.011**	7/42 (16.7%)	2/42 (4.8%)	.156
Surgical site infection	0 (0%)	0 (0%)	‐	0 (0%)	0 (0%)	‐
Fascia dehiscence	2 (1.3%)	0 (0%)	‐	1 (2.3%)	0 (0%)	‐
Deep vein thrombosis	1 (0.6%)	0 (0%)	‐	0 (0%)	0 (0%)	
Pleural effusion	0 (0%)	0 (0%)	‐	0 (0%)	0 (0%)	
Pneumonia or atelectasis	3 (2.1%)	1 (2.4%)	.231	1 (2.4%)	1 (2.4%)	.417
Urinary tract infection	0 (0%)	0 (0%)	‐	0 (0%)	0 (0%)	
Intra‐abdominal sepsis	0 (0%)	0 (0%)	‐	0 (0%)	0 (0%)	
Central vein line sepsis	1 (0.6%)	0 (0%)	‐	1 (2.4%)	0 (0%)	
Ascites	6 (4.1%)	0 (0%)	‐	1 (2.4%)	0 (0%)	‐
Pulmonary embolism	2 (1.3%)	0 (0%)	‐	0 (0%)	0 (0%)	‐
Postoperative bleeding	1 (0.6%)	0 (0%)	‐	1 (2.4%)	0 (0%)	‐
Acute kidney injury	4 (2.7%)	1 (2.4%)	.284	0 (0%)	1 (2.4%)	.222
Fluid collection in resection surface	2 (1.3%)	0 (0%)	‐	0 (0%)	0 (0%)	‐
Abscess in resection surface	4 (2.7%)	0 (0%)	‐	1 (2.4%)	0 (0%)	‐
Bile leak	4 (2.7%)	0 (0%)	‐	1 (2.4%)	0 (0%)	‐
Liver failure	0 (0%)	0 (0%)	‐	0 (0%)	0 (0%)	‐
Aspiration and cardiac arrest	1 (0.6%)	0 (0%)	‐	0 (0%)	0 (0%)	‐
Length of stay, days mean ± SD	6.7 ± 6.6	3.4 ± 2.2	**<.001**	6.6 ± 5.4	3.4 ± 2.2	**.001**
90‐day mortality *n* (%)	0/146 (0%)	0/42 (0%)	‐	0/42 (0%)	0/42 (0%)	‐

*Note*: Before‐PSM all patients before propensity score matching. After‐PSM after propensity score matching. Abbreviation: CD, Clavien‐Dindo classification; OLS, open liver surgery; PSM, propensity score matching. Data are expressed as mean ± SD or as number (percentage). *p* < .05 was considered statistically significant. Bolded values indicates statistically significant differences.

Subgroup analyses of perioperative outcomes for patients undergoing minor resection are summarized in Table 4. After PSM, OT tended to be significantly longer after RLS (230.2 vs. 157.0 min, *p* < .001). In terms of EBLs, the RLS group had significantly fewer EBLs than the OLS group (178.3 vs. 1209.5 mL, *p* = .006), and there was a trend toward lower intraoperative transfusion rates, but no significant difference between the two groups (0% vs. 27.3%, *p* = .218). The overall complication rate, including biliary fistula and pneumonia, were significantly lower for RLS (0% vs. 16.1%, *p* = .022), although minor and major complications were comparable between the two groups. LOS for RLS tended to be significantly shorter than for OLS (3.4 vs. 6.5 days, *p* = .006). There was no 90‐day mortality between the two groups.

**TABLE 4:** Perioperative outcomes: comparison between open and robotic liver surgery in minor resections in patients ≥70 years.

Variables	Before‐PSM		*p*‐value	After‐PSM		*p*‐value
	Open liver surgery (*n* = 106)	Robotic liver surgery (*n* = 33)		Open liver surgery (*n* = 31)	Robotic liver surgery (*n* = 33)	
Operative time min mean ± SD	168.5 ± 59.1	230.2 ± 102.0	**.002**	157.0 ± 56.0	230.2 ± 102.0	**.001**
Estimated blood loss mL mean ± SD	843.7 ± 843.1	148.9 ± 137.7	**<.001**	683.5 ± 564.3	148.9 ± 137.7	**<.001**
Transfusion, *n* (%)	18/106 (17.0%)	2/33 (6.1%)	.159	4/31 (12.9%)	2/33 (6.1%)	.491
Resection margins for malignant lesions, R1	0/106 (0%)	3/33 (9.1%)	.012	0/31 (0%)	3/33 (9.1%)	.239
CD I/II, *n* (%)	13/106 (12.3%)	0/33 (0%)	**.038**	2/31 (6.5%)	0/33 (0%)	.231
CD III/IV, *n* (%)	5/106 (4.7%)	0/33 (0%)	.339	3/31 (9.7%)	0/33 (0%)	.108
All complications *n* (%)	18/106 (17.0%)	0/33 (0%)	**.007**	5/31 (16.1%)	0/33 (0%)	**.022**
Surgical site infection	0	0		0	0	
Fascia dehiscence	0	0		0	0	
Deep vein thrombosis	1	0		0	0	
Pleural effusion	0	0		0	0	
Pneumonia or atelectasis	3	0		1	0	
Urinary tract infection	0	0		0	0	
Intraabdominal sepsis	0	0		0	0	
Central vein line sepsis	1	0		1	0	
Ascites	3	0		0	0	
Pulmonary embolism	0	0		0	0	
Postoperative bleeding	1	0		1	0	
Acute kidney injury	4	0		0	0	
Fluid collection in resection surface	1	0		0	0	
Abscess in resection surface	2	0		1	0	
Bile leak	2	0		1	0	
Liver failure	0	0		0	0	
Aspiration and cardiac arrest	0	0		0	0	
Length of hospital stay (days)	5.8 ± 4.5	3.4 ± 2.4	**.003**	6.5 ± 5.4	3.4 ± 2.4	**.006**
90‐day mortality	0/106 (0%)	0/33 (0%)	‐	0/31 (0%)	0/33 (0%)	‐

*Note*: Data are expressed as mean ± SD or as number (percentage). Abbreviation: CD, Clavien‐Dindo classification; PSM, propensity score matching. *p* < .05 was considered statistically significant. Bolded values indicates statistically significant differences.

Subgroup analyses of perioperative outcomes for patients undergoing major resection are summarized in Table 5. After PSM, OT was significantly longer for RLS compared with OLS (353.2 vs. 188.0 min, *p* < .001). There was a trend toward significantly less EBL for RLS (178.3 vs. 1209.5 mL, *p* = .006) and a trend toward lower intraoperative blood transfusion rates for RLS, but no significant difference between the two groups (0% vs. 27.3%, *p* = .218). Minor, major, and overall complication rates were statistically similar between the two groups. Mean LOS for RLS tended to be significantly shorter than for OLS, but the difference was not significant (3.6 vs. 6.8 days, *p* = .083). There was no 90‐day mortality between the two groups (Table 5).

**TABLE 5:** Perioperative outcomes: comparison between open and robotic liver surgery in major resections in patients ≥70 years.

Variables	Before‐PSM		*p*‐value	After‐PSM		*p*‐value
	Open liver surgery (*n* = 40)	Robotic liver surgery (*n* = 9)		Open liver surgery (*n* = 11)	Robotic liver surgery (*n* = 9)	
Operative time min mean ± SD	206.3 ± 60.2	353.2 ± 99.9	**.002**	188.0 ± 47.0	353.2 ± 99.9	**.001**
Estimated blood loss mL mean ± SD	1392.3 ± 1198.0	178.3 ± 181.9	**<.001**	1209.5 ± 970.7	178.3 ± 181.9	**.006**
Transfusion, *n* (%)	12/40 (30.0%)	0/9 (0%)	.090	3/11 (27.3%)	0/9 (0%)	.218
CD I/II, *n* (%)	7/40 (17.5%)	1/9 (11.1%)	1.000	1/11 (9.1%)	1/9 (11.1%)	1.000
CD III/IV, *n* (%)	6/40 (15.0%)	1/9 (11.1%)	1.000	1/11 (9.1%)	1/9 (11.1%)	1.000
All complications *n* (%)	13/40 (32.5%)	2/9 (22.2%)	.702	2/11 (18.2%)	2/9 (22.2%)	1.000
Surgical site infection	0	0		0	0	
Fascia dehiscence	2	0		1	0	
Deep vein thrombosis	0	0		0	0	
Pleural effusion	0	0		0	0	
Pneumonia or atelectasis	0	1		0	1	
Urinary tract infection	0	0		0	0	
Intra‐abdominal sepsis	0	0		0	0	
Central vein line sepsis	0	0		0	0	
Ascites	3	0		1	0	
Pulmonary embolism	2	0		0	0	
Postoperative bleeding	0	0		0	0	
Acute kidney injury	0	1		0	1	
Fluid collection in resection surface	1	0		0	0	
Abscess in resection surface	2	0		0	0	
Bile leak	2	0		0	0	
Liver failure	0	0		0	0	
Aspiration and cardiac arrest	1	0		0	0	
Length of hospital stay (days) mean ± SD	9.0 ± 10.0	3.6 ± 1.4	.115	6.8 ± 5.5	3.6 ± 1.4	.083
90‐day mortality *n* (%)	0/40 (0%)	0/9 (0%)	‐	0/11 (0%)	0/9 (0%)	‐

*Note*: Data are expressed as mean ± SD or as number (percentage). Abbreviation: CD, Clavien–Dindo classification; PSM, propensity score matching. *p* < .05 was considered statistically significant. Bolded values indicates statistically significant differences.

## 4 DISCUSSION

In recent years, the feasibility and safety of LLS in elderly patients with liver tumors has been evaluated showing favorable results.^7^, ^8^, ^9^ There are few reports currently available regarding RLS in elderly patients. Our previous study found that RLS in patients ≥75 years had similar short‐term outcomes compared to younger patients, especially if perioperative risks are comparable. This study justifies safety of RLS in patients ≥75 years; however, it has not yet been compared to OLS.^16^ The aim of this study was to investigate the impact of RLS and OLS on short‐term perioperative outcomes in patients ≥70 years undergoing liver resection at the High Volume HPB Center in Denmark. The results showed that after PSM, RLS was the preferential approach for elderly patients with improved short‐term outcomes in terms of blood loss and length of hospital stay (Table 3). Furthermore, in patients undergoing minor liver resection, RLS was associated with less blood loss, lower postoperative complication rates, and shorter hospital stay (Table 4). These results are in line with previous reports of LLS in the elderly and support the benefits of RLS in reducing blood loss, decreasing the incidence of postoperative complications, and shortening hospital stay.^7^, ^8^, ^9^

In relation to the number of RLS during our study period, Denmark has a public healthcare system which has a guaranteed waiting time. Unfortunately, there are capacity issues and long waiting times for access to the robotic platform. Therefore, some patients require OLS even if RLS is available and the capacity to offer RLS is currently limited to 10%–15% of all liver resections at our institution.

One strength of RLS is minimal intraoperative blood loss. After PSM, EBL was significantly lower for RLS (155.2 mL for RLS vs. 821.2 mL for OLR, *p* < .001; Table 3). There was, however, a trend toward fewer patients requiring transfusion for RLS (4.8% for RLS vs. 16.7% for OLR, *p* = .156; Table 3). These results were similar to previous reports on LLS in the elderly.^7^, ^8^, ^9^ Several factors may have contributed to less bleeding for RLS. First, RLS is superior to standard laparoscopic surgery due to the special characteristics of the robotic platform, namely tremor filtering, motion scaling, and magnified 3D vision, which enhance the precision of the surgical movements, especially when operating in confined spaces. Furthermore, the articulated motion of the instruments provides superior performance in vascular control, hemostasias, and liver parenchymal resection.^14^ Second, careful fluid management and maintenance of low central venous pressure (CVP) are usually used during liver resection to reduce intraoperative blood loss; with the robotic approach, pneumoperitoneal pressure and CVP can be controlled to stabilize hemodynamics and minimize bleeding.^14^

Postoperative pulmonary complications are associated with significant morbidity after liver surgery, occurring in 17% to 20% of patients and can prolong hospital stay. Furthermore, it is important to recognize that, in the elderly, this is a potentially life‐threatening complication following liver resection. ^7^, ^17^ RLS using insufflation pressure has been associated with decreased pulmonary compliance, venous return, intra‐abdominal organ vascular perfusion, and increased risk of adverse events such as gas embolization rates, and cardiopulmonary complications and acute kidney injury.^18^, ^19^ However, in the LLR report in elderly patients by Nomi et al.,^20^ the incidence of major complications and postoperative pulmonary and cardiovascular complications were significantly less frequent for LLR than for OLS (*p* < .001, *p* = .008, and *p* = .014, respectively). In a similar study, Delvecchio et al.^21^ compared LLS and OLS using propensity score matched analysis in a multicenter study. The incidence of pulmonary complications was significantly lower for LLS than for OLS (4% vs. 12%, *p* = .01), and in conclusion, they report that pure laparoscopic surgery reduced postoperative pulmonary complications in elderly patients (≥70 years). Badawy et al.^22^ also performed a PSM analysis comparing the surgical outcomes of liver resection by LLS and OLS in elderly patients and concluded that minor liver resection in LLS is safe and feasible, and that it is associated with less blood loss, shorter hospital stays, fewer postoperative complications, and better oncological outcomes. In our study, he incidence of postoperative complications after PSM tended to be lower in the RLS group, but the difference was not significant (4.8% for RLS vs. 16.7% for OLR, *p* = .156: Table 3). In terms of the incidence of postoperative pulmonary complications, there was also no statistically significant difference between the two groups (2.4% for RLS vs. 2.4% for OLR, *p* = .417: Table 3). In an analysis of minor liver resection in subgroups, the incidence of postoperative complications after PSM was significantly lower after RLS (0% for RLS vs. 16.1% for OLR, *p* = .022: Table 4). However, pulmonary complications could not be studied due to the low incidence between the two groups. In analyses of postoperative complication rates and pulmonary complications in major liver resection, no significant differences were found between the two groups. These results suggested that, as reported by Badawy et al.,^22^ our study demonstrated an advantage of RLS over OLS in minor liver resection for elderly patients regarding the overall complication rate. However, the study did not demonstrate a further advantage of RLS over OLS in relation to postoperative pulmonary complications. This was owing to the very low incidence of pulmonary complications between the two groups. Our results are in contrast to those reported by Nomi et al.^20^ and Delvecchio, et al.^21^ Further prospective studies regarding pulmonary complications are therefore warranted.

R status is one of the most important postoperative findings, affecting recurrence‐free and overall survival. In our results, RLS tended to have a significantly higher R1 at the resection margin before PSM (7.1% vs. 0.0%, *p* = .011), but there was no significant difference between the two groups after PSM (7.1% vs. 0%, *p* = .241). In terms of tumor size, the results showed that significantly more large tumor lesions were resected in RLS compared with OLS (42.8 mm vs. 30.9 mm, *p* < .040). The following factors may have contributed to the high trend for R1 resection in RLS. First, we consider that the advantages of the robotic system may also contribute to surgeons daring to perform robotic surgery with larger lesions. A most discussed limitation of the robot‐assisted approach is probably the lack of tactile sensation when compared to conventional open surgery. In order to achieve an R0 resection, the essential factors are to maintain a safe surgical margin, dissect the liver parenchyma consistently and not move away from the predefined resection plane. Many robotic surgeons perform liver parenchymal dissection based on the advantages of the robotic system and visual cues, but the lack of tactile sensation may affect the condition of the resection margin. Second, R1 resection was identified in 7.1% (3/42) in RLS, all of whom were aged 75 years or more. An important question remains whether major hepatectomy is desirable in patients ≥75 years. One of the concerns regarding major hepatectomy in the elderly is the metabolic and physiological reserve of the liver and therefore its regenerative capacity. These factors reduce the liver's functional reserves in the elderly, thus predisposing patients to postoperative liver failure. Selective portal vein embolization or two‐stage liver resection is therefore widely used as a measure of choice to regenerate and expand the remaining liver. However, in our opinion, parenchymal‐sparing liver resection is also a useful strategy to avoid postoperative liver failure, especially in high‐risk patients (patients with reduced liver function, elderly patients with comorbidities). These results may be due to the fact that our center always considers the risk of postoperative liver failure in elderly patients and recommends avoiding major liver resection and opting instead for parenchymal‐sparing liver resection.

With respect to operative time, RLS, like LLS, is a more complex and time‐consuming procedure than conventional OLS. In the literature, longer operative time is associated with early postoperative complications.^17^, ^23^, ^24^ There are two main reasons for longer operative time in RLS: one is the time required to dock the robotic device to the patient; the second is the limitation of the robotic device used for liver parenchymal dissection, which requires frequent changes of the robotic arm's instruments.^13^ In a multicenter study by Di Benedetto et al.^25^ using PSM comparing RLS and OLS, they reported that operative time was significantly longer in the RLS group compared to the OLR group (median [IQR], 295 [190–370] min vs. 200 [165–255] min, docking included *p* < .001). RLS operative time was significantly longer than OLS (256.6 min for RLS vs. 165.1 min for OLR, *p* < .001; Table 3). The results of our study are similar to those of Di Benedetto et al.^25^ and are considered valid. There is also a focus on the use of instrumentation, particularly the absence of CUSA in robotic surgical instruments. In our previous study, we discussed robotic devices used for liver parenchymal dissection and the discussion regarding a fully robotic approach or a hybrid approach using CUSA.^14^ There is still considerable room for reducing RLS operative time, which will depend on future innovations in robotic surgical instruments.

According to previous data, an important advantage of minimally invasive surgery is a faster functional recovery. Indeed, minimally invasive surgery has the advantage of shorter hospital stays compared to OLS, due to a lower rate of postoperative complications and a faster return to daily activities.^5^, ^6^, ^7^, ^8^, ^9^ In general, elderly patients tend to have longer postoperative hospital stays than younger patients. It is therefore essential for elderly patients to be rehabilitated as quickly as possible to restore postoperative function before being discharged home. All patients in our hospital are treated according to the ERAS program, and perioperative care after liver surgery is based on early discharge from hospital.^15^ In RLS, surgical wall trauma is dramatically reduced as the resected specimen is removed through a limited number of incisions (6 to 7). This reduction in surgical trauma is expected to reduce acute reactions, reduce postoperative pain, and restore pulmonary function in the early postoperative period. The above advantages lead to early rehabilitation for the elderly, resulting in shorter discharge compared with OLS (3.4 days for RLS and 6.6 days for OLS *p* = .001; Table 3). These results are consistent with previous studies on elderly patients that showed faster recovery and shorter hospital stays after LLS.^7^, ^8^, ^9^, ^20^, ^21^, ^22^

One criticism that has hindered the widespread use of robotic surgery is that it is considerably more expensive. However, reports by Sham et al.^26^ and Daskalaki et al.^27^ show that length of hospital stay is significantly shorter for RLS; furthermore, intraoperative blood loss is lower, which reduces the need for blood transfusions, postoperative analgesia and antibiotics, resulting in lower final total costs. A shorter length of hospital stay is an important factor with a direct socioeconomic impact on physical healing, social reintegration, and total surgical costs. Most importantly, lower postoperative complication rates ultimately reduce total costs. Although our study did not perform a cost comparison or cost‐effectiveness analysis, the average difference in hospital stays of 3 days represents significant savings for the hospital (Table 3).

The main limitation of our study was its retrospective nature. Even though we adjusted to reduce bias by matching patients according to critical criteria, prospective randomized trials of OLS versus RLS are needed to confirm the safety and efficacy of RLS in elderly patients with liver tumors. Second, studies did not assess the preoperative risk, and most of the included studies did not report comorbidities.

## 5 CONCLUSION

The use of the robotic approach in patients over 70 years of age undergoing liver resection was associated with reduced blood loss and shorter hospital stay. In particular, robotic minor liver resection is associated with significantly reduced postoperative morbidity as compared to the open approach. We therefore conclude that age is not a contraindication for RLS and it should be considered as a safe alternative to OLS in elderly patients. However, further randomized controlled studies are needed to establish firm evidence for the role of the robotic approach in the elderly population undergoing liver surgery.

## AUTHOR CONTRIBUTIONS

The authors confirm their contributions to the study as follows: Study conception and design: Daisuke Fukumori, Christoph Tschuor, Takashi Hamada, Luit Penninga, Jens Hillingsø and Peter Nørgaard Larsen. Data collection: Daisuke Fukumori. Analysis and interpretation of results: Daisuke Fukumori, Christoph Tschuor and Luit Penninga. Draft manuscript preparation: Daisuke Fukumori. All authors reviewed the results and approved the final version of the manuscript.

## FUNDING INFORMATION

This research did not receive any specific grant from funding agencies in the public, commercial, or not‐for‐profit sectors. Due to the fact that this study was a retrospective quality control‐based study, no ethical committee approval was required.

## CONFLICT OF INTEREST STATEMENT

The authors confirm that there are no conflicts of interest.

## Data Availability

The data that support the findings of this study are available from the corresponding author upon reasonable request.
